# Low Protein Intake Is Associated with Frailty in Older Adults: A Systematic Review and Meta-Analysis of Observational Studies

**DOI:** 10.3390/nu10091334

**Published:** 2018-09-19

**Authors:** Hélio José Coelho-Júnior, Bruno Rodrigues, Marco Uchida, Emanuele Marzetti

**Affiliations:** 1 Applied Kinesiology Laboratory–LCA, School of Physical Education, University of Campinas, Av. Érico Veríssimo, 701, Cidade Universitária “Zeferino Vaz”, Barão Geraldo, Campinas-SP 13083-851, Brazil; prof.brodrigues@gmail.com (B.R.); uchida@g.unicamp.br (M.U.); 2 Department of Geriatrics, Neurosciences and Orthopedics, Teaching Hospital “Agostino Gemelli”, Catholic University of the Sacred Heart, 00168 Rome, Italy; emarzetti@live.com

**Keywords:** frailty, protein intake, older adults

## Abstract

(1) Background: Several factors have been suggested to be associated with the physiopathology of frailty in older adults, and nutrition (especially protein intake) has been attributed fundamental importance in this context. The objective of this study was to conduct a systematic review and meta-analysis to investigate the relationship between protein intake and frailty status in older adults. (2) Methods: A search of scientific studies was conducted in the main databases (Medline, Scopus, Cochrane library), and in the reference lists of selected articles. The search terms included synonyms and Medical Subject Headings and involved the use of Boolean operators which allowed the combination of words and search terms. Observational studies—cross-sectional and longitudinal—that met the eligibility criteria were included in the review. Article selection and data extraction were performed by two independent reviewers. Meta-analyses with random effects were performed. Publication bias was measured using the Strengthening the Reporting of Observational Studies in Epidemiology instrument. (3) Results: In the final sample, 10 articles, seven cross-sectional and three longitudinal, were included in the present study. Overall, studies investigated a total of 50,284 older adults from three different continents between 2006 and 2018. Four cross-sectional studies were included in the meta-analyses. The results demonstrated that a high protein intake was negatively associated with frailty status in older adults (odds ratio: 0.67, confidence interval = 0.56 to 0.82, *p* = 0.0001). (4) Conclusions: Our findings suggest that a high consumption of dietary protein is inversely associated with frailty in older adults.

## 1. Introduction

The aging process is a continuous phenomenon characterized by alterations in major physiological systems, accompanied by the development of chronic diseases and geriatric syndromes, such as frailty. Frailty may be conceptualized as a multidimensional geriatric clinical state that involves multiple signs and symptoms leading to extreme vulnerability to stressors and resulting in increased risk of negative health-related outcomes (e.g., functional decline, disability, falls, hospitalization, institutionalization, death) [1,2].

Nutrition is acknowledged as a major factor in the context of frailty. In fact, malnutrition is considered one of the pillars for the development of this condition [3], since it can influence all diagnostic criteria for frailty (i.e., unintentional weight loss, low muscle strength, exhaustion, reduced physical activity levels, and slow walking speed) [4]. Three previous systematic reviews have been conducted on the association between nutrition and frailty. Authors observed that several factors might be responsible for this close relationship between frail and nutrition, including oral health, nutritional status, dietary patterns, diet quality, the antioxidant capacity of the diet, micronutrients and macronutrients intake [3,5]. Nevertheless, protein intake might be the main factor behind this relationship, through its actions on muscle mass and strength.

Indeed, human skeletal muscle protein turnover comprises the process of muscle protein synthesis and muscle protein breakdown [6,7,8]. On one hand, muscle hypertrophy occurs when the rates of protein synthesis exceed protein breakdown, which may be elicited by hyper amino acidemia induced by dietary protein intake; on the other hand, an inadequate protein intake leads to lower protein synthesis rate, resulting in net protein breakdown and muscle catabolism [6,7,8]. During aging, numerous process collaborate to a reduced protein intake, such as lack of hunger, impaired oral health, and loss of acuity in taste, smell and sight, to quote a few [9]; consequently, collaborating to muscle catabolism [9]. In addition, evidence has demonstrated that the anabolic response to hyper aminoacidemia may be blunted in older adults [10,11], which indicate that this population should consume larger amounts of protein in comparison to young adults in an attempt to maintain muscle protein synthesis. Nevertheless, over time, the lack of adequate protein intake leads to a state called as sarcopenia [9,12,13], which is characterized by marked muscle atrophy, dynapenia, and reduced physical function, all variables encompassed on frailty definition [14]. If there is no immediate intervention to reduce sarcopenia and frailty progression, as well as improve protein intake, the patients will develop a severe physical disability and consequently exhaustion and sedentary behavior [1,15]. 

It should be stressed that other pathways besides sarcopenia may be also responsible for the association between protein intake and frailty, since evidence has demonstrated that protein intake is associated with dementia, global cognitive scores, visuospatial skill, nonverbal memory, and logical memory in older adults [16,17,18]; all aspects linked with frailty [1].

However, investigations on the association between protein intake and frailty have shown positive, negative and even null results. In addition, to the best of our knowledge, there is a lack of systematic reviews and meta-analysis dedicated to investigating the relationship between protein intake and frailty in older adults. 

Therefore, the present study was conducted to perform a systematic review to identify and compare studies reporting the relationship between frailty status and protein intake in older adults. Additionally, data were combined to calculate the pooled overall relationship between frailty status and protein intake.

## 2. Materials and Methods 

We conducted a systematic review and meta-analysis of observational studies to investigate and quantify the association between protein intake and frailty in older adults. The study was fully performed by investigators and no librarian was part of the team. This study complies with the criteria of the Primary Reporting Items for Systematic Reviews and Meta-analyses (PRISMA) Statement [19] and the Meta-analysis of Observational Studies in Epidemiology (MOOSE) guidelines [20]. 

### 2.1. Eligibility Criteria

The inclusion criteria of the present study consisted of: (a) observational studies, including cross-sectional, case-control and longitudinal studies, which investigated as primary or secondary outcome the association of protein intake and frailty in older adults; (b) study sample 60 years or older; (c) frailty defined by a validated scale; (d) reported information on the proportion of frailty among those with high and low levels of protein intake; (e) published studies (English language). To be included in the meta-analysis, in addition to the aforementioned inclusion criteria, the investigations had to provide: (f) at least two groups divided according to protein intake (e.g., high and low), (g) the prevalence of frailty in each group, (h) and the total sample size in each group. We excluded randomized-clinical trials (RCTs), quasi-experimental, cross-over studies and any kind of investigation which examined the effects of a nutritional intervention associated or not with other interventions (e.g., physical exercise) on frailty. Studies that classified the volunteers as frail according to reduced physical/or cognitive function were also excluded.

### 2.2. Search Strategy and Selection Criteria

Studies published on or before July 2018 were retrieved from the following three electronic databases by one investigator: (1) PubMed, (2) the Cochrane Library, and (3) SCOPUS. Reference lists for reviews and retrieved articles for additional studies were checked and citation searches on key articles were performed on Google Scholar and ResearchGate for additional reports. Initially, a search strategy was designed using keywords, MeSH terms, and free text words such as protein intake, frailty, older adults. Additionally, keywords and subject headings were exhaustively combined using Boolean operators. The complete search strategy used for the PubMed can be shown in List S1. Only eligible full texts in English language were considered for review. Authors were contacted if necessary.

### 2.3. Data Extraction and Quality Assessment 

Titles and abstracts of retrieved articles were screened for eligibility by two researchers. If an abstract did not provide enough information for evaluation, the full-text was retrieved. Disagreements were solved by a third reviewer. Reviewers were not blinded to authors, institutions, or manuscript journals. Data extraction was independently performed by two reviews using a standardized coding form. Disagreements were solved by a third reviewer. Coded variables included methodological quality and the characteristics of the studies. The quality of reporting for each study was performed by two researchers using the Strengthening the Reporting of Observational Studies in Epidemiology (STROBE) instrument [21]. The agreement rate between reviewers was κ = 0.98 for quality assessment.

### 2.4. Statistical Analysis 

The meta-analysis was conducted using Revman V.5. Effect sizes (ESs) were measured using odds ratio (OR) and 95% confidence intervals (CIs). The OR indicates the risk for frailty according to protein intake, high in relation to low. A significant OR is required to have a 95% confidence interval (CI 95%) that did not include the value of 1 and a *p* value for the test of significance of the total overall effect (Z) lower than 0.05. An inverse variance random-effect model was used to calculate the pooled ES since the studies demonstrated different characteristics regarding the main aspects associated with frailty (e.g., modified frailty criteria), protein intake (e.g., different cut-offs for high and low protein intake definition), and covariates (e.g., energy intake). Funnel plots and Egger’s regression analysis were used to evaluate the publication bias. Heterogeneity across studies was tested using the Q-statistics and *I²* index was used to assess inconsistency [22]. Additionally, *I²* index was classified as might not be important (0–40%), may represent moderate heterogeneity (30–60%), may represent substantial heterogeneity (50–90%), and considerable heterogeneity (75–100%) [22]. Forest plots were used to illustrate summary statistics and the variation (heterogeneity) across studies.

## 3. Results

### 3.1. Literature Search

**Figure 1:**
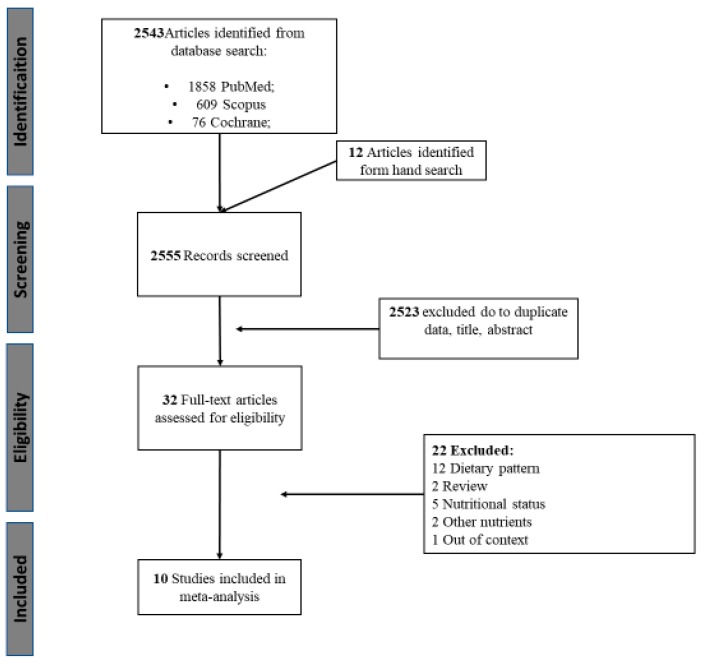
Flow chart of the present study.

Of the 2555 registers recovered from electronic databases and hand search, 2523 records were excluded based on duplicate data, title or abstract. Thirty-two studies were fully reviewed and assessed for eligibility. Finally, 10 studies met the inclusion criteria (Figure 1).

### 3.2. Characteristics of the Included Studies

Table 1 provides a general description of the included studies. Overall, a total of 18,120 community-dwelling older adults from five different countries (France, Germany, Italy, Japan, and the United States of America) were investigated between 2006 and 2018 in the cross-sectional studies. Frailty assessment was performed with two tools. The frailty phenotype proposed by Fried et al. (2001) was used in six of the seven studies [23,24,25,26,27,28], while one study used the Kihon checklist (KCL) [29]. However, it is important to mention that the frailty phenotype [14] was modified in 5 of the 6 studies. Indeed, weight loss criterion was modified in the studies of Rahi et al. [28] and Shikany et al. [27], while Bartali et al. [23] removed this variable. In turn, in the investigations performed by Kobayashi et al. [24,25], slowness and weakness were indirectly measured based on a questionnaire. Slowness assessment was also modified in the study of Rahi et al. [28]. Dietary intake was primarily assessed by population-specific food-frequency questionnaires (FFQ) (57.1%) [23,26,27], followed by self-administered diet history questionnaires (28.6%) [24,25], and the 24 h dietary recall (14.3%) [28]. High and low protein intake was differently defined in the investigations. Measures of centrality (e.g., tertiles, quartiles, quintiles) were used in 6 of the 7 studies [23,24,25,26,27], while Rahi et al. [28] performed the analysis based on a pre-established cut-off (i.e., protein intake levels ≥ 1 g/kg of body weight). Regarding longitudinal studies, 32,164 community-dwelling older adults were investigated between 2010 and 2016. The studies were conducted in North America (United States of America) and Europe (Spain). The mean duration of follow-up was 3.7 years (3.0–4.6 years). The frailty phenotype was used in all studies for frailty assessment. However, as was observed in cross-sectional studies, the frailty phenotype was modified in 2 of the 3 longitudinal studies. Shikany et al. [27] considered the loss of appendicular lean mass as a measurement of weight loss. In turn, Beasley et al. [30] used a modified version of frailty phenotype as they measured muscle weakness and slowness using the Rand-36 Physical function scale. FFQ (66.6%) and computerized face-to-face diet history (33.3%) were used for a dietary intake assessment. In longitudinal studies, all investigations used measures of centrality (i.e., quartile and quintile) to determine the levels of protein intake.

### 3.3. Quality Assessment

The overall score of the quality assessment of cross-sectional and longitudinal studies is shown in Table 1 and the analysis of each variable is detailed in Tables S1 and S2, respectively. The point by point analysis is shown in Table S3. The overall score of cross-sectional studies ranged from 19 to 22. All studies reported the items required by the STROBE criteria in relation to the abstract (items 1 and 2), objectives and hypothesis (items 3 and 4), described the settings, locations, relevant dates, eligibility criteria and the source and methods of selection of participants (items 5 and 6), clarity of the outcomes (items 7), methods of assessment (item 8), handle of the quantitative variables (item 11), give the characteristics of study participants (item 14), reported the number of outcome events (item 15), statistical methods and analysis (items 12, 16, 17), and discussion (items 18–21).However, 57.1% of the studies failed to clearly report the efforts performed to address potential sources of bias (item 9) [24,26,27,28], 42.9% did not properly explain how the study size arrived at (item 10) [26,27,28], and 14.3% did not show the number of individuals at each stage of study (item 13) [26].

Similar results were seen in longitudinal studies, in which all investigations received a STROBE score of 20. None of the studies adequately presented a description of how the study was arrived at (item 10), while 66.6% failed to describe any efforts to address potential sources of bias (item 9) [27,31], and 33.3% did not show the number of individuals at each stage of study (item 13) [30].

### 3.4. Association between Protein Intake and Frailty

#### 3.4.1. Protein Intake and Frailty Prevalence (i.e., Cross-Sectional Studies)

**Figure 2:**
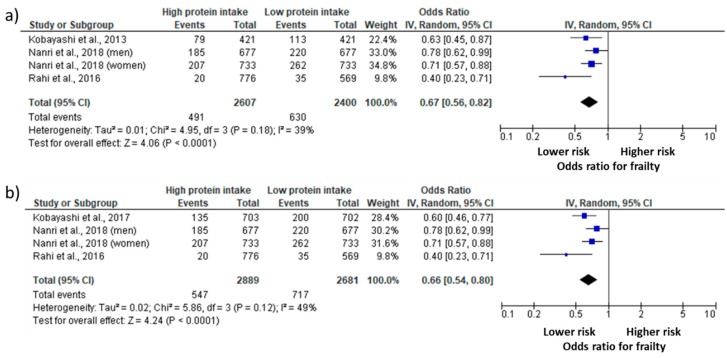
Odds ratio (OR) of the prevalence of frailty in older adults with high and low protein intake. Squares represent study-specific estimates; diamonds represent pooled estimates of random-effects meta-analyses. (**a**) The analysis was performed included Kobayashi et al. 2013; (**b**) The analysis was performed included Kobayashi et al. 2017.

A total of four studies provided information regarding different intakes of protein in at least two groups, the prevalence of frailty in each group, and the total sample size in each group; therefore, they were added in the meta-analysis (Figure 2). Two aspects should be mentioned before the presentation of data. First of all, Nanri et al. [29] provided the data according to gender, and the results are presented accordingly. In turn, the investigations performed by Kobayashi et al. [24,25] used the same database (i.e., Three-generation Study of Women on Diets and Health), so that the studies were not analyzed in combination. The overall meta-analysis results showed a 0.67 OR (Figure 2a) and a 0.66 OR (Figure 2b) for frailty (95% CI = 0.56 to 0.82, *p* = 0.0001; 95% CI = 0.54 to 0.80, *p* = 0.0001) in older adults with high protein intake compared with low protein intake according to the inclusion of Kobayashi et al. [24] or Kobayashi et al. [25], respectively. When the study of Kobayashi et al. [25] was not in the analysis, it was possible to observe an *I²* lower than 40% accompanied by a *p* = 0.18, indicating that this heterogeneity might not be important [22]. However, when the study of Kobayashi et al. [24] was removed, the *I²* increased to 49% and *p* value was of 0.12, which can indicate a moderate heterogeneity [22].

**Figure 3:**
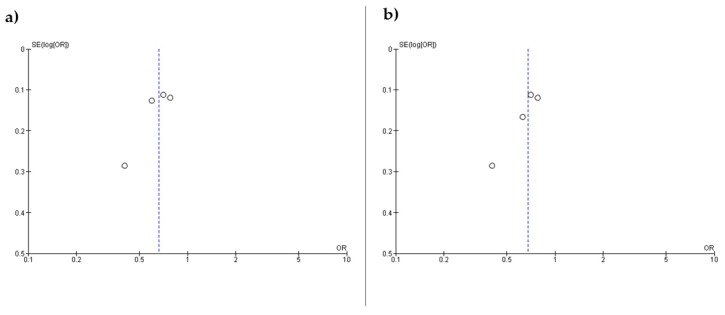
Funnel plots including (**a**) Kobayashi et al. 2013 and (**b**) Kobayashi et al. 2017 OR.

Figure 3 shows the funnel plots (a) and (b) based on the primary outcome according to the inclusion of Kobayashi et al. [24] or Kobayashi et al. [25], respectively. The figures are asymmetrical indicating that potential publication bias might influence the results of this review. Egger’s linear regression test indicated possible publication bias for the association when the study of Kobayashi et al. [24] was included (*p* = 0.02), but not Kobayashi et al. [25] (*p* = 0.09). 

#### 3.4.2. Protein Intake and Frailty Risk (i.e., Longitudinal Studies)

We found three studies that evaluated the longitudinal relationship between protein intake and frailty risk. The findings demonstrate that two of the three studies observed that higher protein intake was negatively associated with frailty risk.

## 4. Discussion

Frailty is a multifactorial condition associated with poor prognosis. Low protein intake has been proposed among the factors possibly involved in the pathogenesis of frailty. We, therefore, performed a systematic review and meta-analysis to investigate the relationship between protein intake and frailty in older adults. The main findings of the present study indicate that low protein intake is associated with frailty prevalence in older adults.

Study quality assessment demonstrated that reports were of very good quality, such that cross-sectional studies scored between 19 and 22 and all longitudinal studies scored 20. Interestingly, cross-sectional and longitudinal studies did not provide the same items, including efforts to address potential sources of bias (item 9), the design of the study size (item 10), and the report regarding the number of participants in all the phases of the study (item 13). 

Some recent systematic and descriptive reviews have investigated the relationship between nutrition and frailty [3,4,32,33]. However, none of these studies was specifically designed to investigate the role of protein intake in this phenomenon and the findings were not quantitatively assessed. Thus, to the best of our knowledge, this is the first systematic review and meta-analysis designed to investigate the relationship between protein intake and frailty in older adults.

The results of the present study may be at least partially explained by the theoretical overlap between sarcopenia and physical frailty [34,35]. Indeed, physical frailty, as measured by the Fried’s criteria [14,36], encompasses features as slowness, weakness, exhaustion, and sedentary behavior, which are strongly associated with the sarcopenia condition [34,35]. Slowness (i.e., slow walking speed) and weakness (i.e., low upper-limb muscle strength), for example, are proposed as diagnostic criteria for sarcopenia by the European Working Group on Sarcopenia in Older Persons (EWGSOP) [15], while exhaustion and sedentary behavior are common consequences of sarcopenia progression [37]. Indeed, Landi et al. [35] suggested that sarcopenia may be envisioned as a central mechanism for the development of physical frailty. In another word, physical frailty may be the final pathway of sarcopenia progression [35]. This idea is further supported by the higher prevalence of sarcopenia in pre-frail and frail older adults when compared to non-frail peers [38,39].

Sufficient protein consumption may cause a shifting on net balance in favor of muscle protein synthesis [7,40]. Protein supplementation *per se* has been shown to prevent the progression of physical decline in frail older adults [30,41]. In addition, protein intake has a key role in the physiological adaptations elicited by the resistance training on the neuromuscular apparatus since a greater muscle protein synthesis is expected when both non-pharmacological therapies are offered in combination [6,7]. Taken together, these findings suggest that sufficient protein intake may reverse or at least prevent functional decline in frail older adults.

However, this kind of inference deserves caution since not all evidence has demonstrated the positive effects of protein supplementation on the sarcopenia aspects associated with frailty, such as muscle mass, muscle strength and physical function [42,43]. Finally, it should be noted that the changes observed after protein supplementation may be different from those observed in response to dietary protein intake.

It is worth mentioning, that our main findings are based on cross-sectional studies and causal extrapolations should be performed carefully. Unfortunately, there were no available data from longitudinal studies to perform a meta-analysis. Overall, findings are still controversial. Shikany et al. [27] observed that protein intake was inversely associated with the risk of transitioning from robust to pre-frail status in a range of 4.6 years, while there were no significant associations between protein and frailty status. However, Sandoval-Insausti et al. [31] reported that total protein and animal protein intake were inversely associated with frailty and its components (i.e., slowness) over a mean follow-up of 3.5 years. Similarly, Beasley et al. [30] concluded that higher protein intake was associated with reduced risk of frailty in community-dwelling older women.

Interestingly, the main variables investigated in the present study were differently defined across the investigations. Regarding frailty, although this variable was assessed using the frailty phenotype in most investigations, adaptations of some of the criteria were observed in 5 of the 6 cross-sectional studies, as well as in 2 of the 3 longitudinal studies. In fact, weight loss criterion was modified in the trial of Rahi et al. [28], in which researchers included volunteers with self-reported unintentional loss > 3 kg or as a body mass index < 21 kg/m^2^, while Shikany et al. [27] included subjects who lost appendicular muscle mass. In turn, Bartali et al. [23] removed the weight loss criterion of their investigation. Slowness and weakness were also modified. In this case, Kobayashi et al. [24,25], Beasley et al. [30], and Rahi et al. [28] (only slowness) used self-reported questionnaires instead of direct evaluations. It is also possible to observe that different cutoffs to define high a low protein intake (i.e., tertiles, quartiles, quintiles and pre-established values) were used in the investigations.

These modifications have direct implications in the findings of the present study. Although scales and questionnaires may offer more information in a shorter period when compared to performance-based measurements, evidence has demonstrated the limited capacity of these tools to reflect different measures of physical status [44,45]. This probably occurs because the results of patient-reported questionnaires may be biased due to mood, motivation, fatigue, health status, fluctuations in memory, and the specific knowledge and familiarity with the questionnaires and scales [44,45]. In this sense, different results than those observed in the present study could occur if the investigations were performance based on direct measures, as proposed by Fried et al. [14]. Furthermore, the use of different cut-offs to define protein intake levels leads to disagreements and restrict the proposal of public health recommendations to older adults due to the range of approaches used by the studies.

Taken together, these differences may also explain the heterogeneity of results observed among the longitudinal studies. Nevertheless, different settings, eligibility criteria, gender, sarcopenia status, dietary assessment methods, and follow-up periods of the various studies may also explain this variability. In this sense, more well-controlled cross-sectional and longitudinal studies are still necessary to improve the actual knowledge about frailty and protein intake in older adults, as well as to confirm our findings.

We should state the absence of subgroup analyses as the major limitation of the present study. Indeed, the use of crude OR limits interpretation of our meta-analysis, since the influence of important covariates (e.g., age, type of protein [animal, vegetal], sarcopenia) were not taken into consideration in the results, and we recommend that readers interpret our results carefully. The main aspect that prevented us to perform the analysis was the lack of available data in the included studies. Regarding dietary assessment, it is worth mentioning that total protein intake, which was used in all studies for comparisons, is probably not the best parameter to represent adequate protein consumption, since investigations in the context of physical function and sarcopenia have used relative protein intake (g/kg/day) [46,47,48]. In addition, recent evidence has demonstrated that a spread distribution of protein intake during the main meals is better associated with gait speed than relative protein intake [49]. Providing support to the importance of the distribution of protein intake, Loenneke et al. [50] observed that a frequent consumption of meals containing at least 30 g of protein was associated with greater lean mass and lower-limb muscle strength in middle-aged and older adults. The role of animal and plant-based protein sources on variables associated with frailty has also been the object of discussion among researchers [51,52]. Therefore, although future investigations are still necessary to confirm our findings, the present study may serve as a guide for future studies in this field; so that investigation should include more information regarding the factors that may interfere in the relationship between protein intake and frailty, taking into account the variables that have been investigated by other studies.

In addition, funnel plots and Egger’s linear regression test indicated that biases from publications and other factors may have had a significant influence on the results of our meta-analysis mainly when the study of Kobayashi et al. [24] was included. Possible explanations for this publication bias included the small number of studies investigated, multiple publication bias, and heterogeneity [22].

Finally, another aspect of the present study that deserves concerns is the use of STROBE instrument as a tool to quality assessment. As discussed by da Costa et al. [53], STROBE was primarily developed to improve the reporting of observational studies. Thus, some may argue that another tool should have been used in the present study. However, it should be stressed that there is no gold standard tool to assess the risk of bias in non-randomized studies, as well as some of the STROBE questions may represent an evaluation of risk of bias; consequently, making it a tool commonly used in systematic reviews and meta-analysis [53].

In conclusion, our findings support the need for increased protein intake in older adults in an attempt to avoid frailty development. 

## Figures and Tables

**Table 1:** General description of the included studies.

Year	Authors	Country	Study Design	Setting	*n*	Mean Age (age range; min–max)	Sex Ratio of Participants (female/male) by frail vs. non-frail	Frailty Assessment Method	Dietary Intake Assessment Method	Protein Intake (g/day)	Protein Intake Level Definition	Outcomes	Covariates Included in Models	Quality Analysis Score
***Cross-sectional***														
2006	Bartali et al. [23]	Italy	Cross-sectional	Community-dwelling	802	74.1	1.2	CHS frailty index (a)	Food-frequency questionnaire	-	Dichotomous	Low protein intake is associated with frailty	Results were adjusted for age, sex, education, economic status, household composition, smoking status, number of diseases, cognitive function, body mass index, and “happiness.”	22
2013	Kobayashi et al. [24]	Japan	Cross-sectional	Community-dwelling	2108	74.7	-	CHS frailty index (b)	Self-administered diet history questionnaire	74.0	Quintile (≤62.9 g/day, 6369.8 g/day, 69.8–76.1 g/day, 76.1–84.3 g/day, ≥84.3 g/day)	Protein intake was inversely associated with frailty	Results were energy-adjusted and for age, BMI, residential block, size of residential area, living alone, current smoking, alcohol drinking, dietary supplement use, history of chronic disease, depression symptoms, and energy intake.	20
2013	Bollwein et al. [26]	Germany	Cross-sectional	Community-dwelling	194	83.0 (75–96)	6.5 vs. 1.3	CHS frailty index	Food-frequency questionnaire	76.6	Quartiles (≤0.90, 0.91–1.07, 1.08, ≥1.27)	Protein intake was not associated with frailty	Results were adjusted for age and sex, instrumental activities of the daily living score, number of medications, and chewing difficulties	19
2014	Shikany et al. [27]	United States of America	Cross-sectional	Commnity-dwelling	5925	75.0	-	CHS frailty index (c)	Food-frequency questionnaire	-	Quintile (≤6.0–13.7%, 13.8–15.2%, 15.3–16.5%, 16.6%–18.3%, 18.4–29.3%)	Protein intake was not associated with frailty	Results were adjusted for age, race, center, education, marital status, smoking, health status, medical conditions, body mass index, and energy intake	20
2016	Rahi et al. [28]	France	Cross-sectional	Community-dwelling	1345	75.6	4.0 vs. 1.46	CHS frailty index (d)	24 h dietary recall	70.4	Dicothomous <1g/kg body weight/day and ≥1g/kg body weight	Protein intake was associated with frailty	The model 1 was adjusted for age, sex, and educational level; and the model 2 was additionally adjusted for BMI, diabetes, cardiovascular history, depression, cognitive performance, number of drugs, and total energy intake.	20
2017	Kobayashi et al. [25]	Japan	Cross-sectional	Community-dwelling	2108	74.0	-	CHS frailty index (b)	Self-administered diet history questionnaire	73.1	Tertile (≤67.6 g/day, 67.6–78.3 g/day, ≥78.3 g/day)	Protein intake was inversely associated with frailty	Dietary total antioxidant capacity	20
2018	Nanri et al. [29]	Japan	Cross-sectional	Community-dwelling	5638	73.2	0.88 vs. 1.05 *	KCL	Food-frequency questionnaire	-	Men = quartiles (≤48.8 g/day, 48.8–56.1 g/day, 56.1–65.4 g/day, >65.4 g/day); Women = quartiles (<43.8 g/day, 43.8–51.1 g/day, 51.1–59.5 g/day, >59.5 g/day)	Protein intake was inversely associated with frailty	For men, the model 1 was adjusted forage, body mass index, total energy intake, alcohol status, smoking status and history of disease and the model 2 was adjusted for family structure, educational attainment, population density, and self-related health.	20
***Longitudinal***														
2010	Beasley et al. [30]	United States of America	Longitudinal (3.0 years follow-up)	Community-dwelling	24,417	65–79	-	CHS frailty index (e)	Food-frequency questionnaire	72.8	Quintiles of protein intake (% kilocalories)	Protein intake was significantly associated with the odds of becoming frail	Results were adjusted for age, ethnicity, BMI, income, education, having a current health care provider, smoking, alcohol, general health status, history of comorbid conditions, history of hormone therapy use, number of falls, whether participant lives alone, disabled defined by at least 1 activity of daily living affected, depressive symptoms, log-transformed calibrated energy intake	20
2014	Shikany et al. [27]	United States of America	Longitudinal (4.6 years follow-up)	Community-dwelling	5925	75.0	-	CHS frailty index (c)	Food-frequency questionnaire	-	Quintile (≤6.0–13.7%, 13.8–15.2%, 15.3–16.5%, 16.6%–18.3%, 18.4–29.3%)	Protein intake was not associated with the odds of becoming frail	Results were adjusted for age, race, center, education, marital status, smoking, health status, medical conditions, body mass index, and energy intake	20
2016	Sandoval-Insausti et al. [31]	Spain	Longitudinal (3.5 years follow-up)	Community-dwelling	1822	68.7	0.9 vs. 2.4	CHS frailty index	Computerized face-to-face diet history	76.6	Quartiles of protein intake	Protein intake was associated with the odds of becoming frail	Results were adjusted for age, energy intake, ethanol, lipids, animal or vegetal protein, level of education, marital status, tobacco consumption, BMI, abdominal obesity, and dietary fiber, diseases.	20

CHS = Cardiovascular Health Study; KCL = Kihon checklist; bw/d = body weight/day; BMI= Body mass index; (a) Bartali et al. used a modified version of the CHS frailty index, since weight loss was removed; (b) Kobayashi et al. used the CHS frailty index version modified by Woods et al as they did not have direct measures of gait speed and strength; (c) Shikany et al., used a modified version of the CHS frailty index as they measured weight loss criterion based on loss of appendicular lean mass; (d) Rahi et al., used a modified version of the CHS frailty index as a loss of 3 kg and a reduced BMI (<21 kg/m^2^) were both accepted as measures of weight loss criterion, slowness was determined based on the Rosow-Breslau test, and weakness was identified using the chair standing method (e) Beasley et al., used a modified version of the CHS frailty index as they measured muscle weakness and slow walking speed using the Rand-36 Physical function scale; * frail vs non-frail.

## Supplementary Materials

The following are available online at http://www.mdpi.com/2072-6643/10/9/1334/s1, List S1. The complete search strategy used for the PubMed, Table S1. Quality assessment of cross-sectional studies, Table S2. Quality assessment of longitudinal studies, Table S3 Point by point analysis.
