# Supplementary material for: Low Protein Intake Is Associated with Frailty in Older Adults: A Systematic Review and Meta-Analysis of Observational Studies
Source: Nutrients. 2018 Sep 19;10(9):1334. doi: 10.3390/nu10091334 (PMC6165078; doi:10.3390/nu10091334)
Supplement: Supplementary file 1 [file nutrients-10-01334-s001.zip › nutrients-339471/List S1.docx]

List S1. Complete search strategy used for the PubMed

1. Protein consumption AND frailty;
2. Protein consumption AND frailty index;
3. Protein consumption AND fragility;
4. Protein intake AND frailty;
5. Protein intake AND frailty index;
6. Protein intake AND fragility;
7. ("proteins"[MeSH Terms] OR "proteins"[All Fields] OR "protein"[All Fields]) AND ("frailty"[MeSH Terms] OR "frailty"[All Fields]);
8. ("proteins"[MeSH Terms] OR "proteins"[All Fields] OR "protein"[All Fields]) AND ("frailty"[MeSH Terms] OR "frailty"[All Fields]) AND (older[All Fields] AND ("adult"[MeSH Terms] OR "adult"[All Fields] OR "adults"[All Fields]));
9. ("proteins"[MeSH Terms] OR "proteins"[All Fields] OR "protein"[All Fields]) AND ("frail elderly"[MeSH Terms] OR ("frail"[All Fields] AND "elderly"[All Fields]) OR "frail elderly"[All Fields] OR ("frail"[All Fields] AND "older"[All Fields] AND "adults"[All Fields]) OR "frail older adults"[All Fields]);
10. ("proteins"[MeSH Terms] OR "proteins"[All Fields] OR "protein"[All Fields]) AND (Kihon[All Fields] AND ("checklist"[MeSH Terms] OR "checklist"[All Fields]));
11. ("proteins"[MeSH Terms] OR "proteins"[All Fields] OR "protein"[All Fields]) AND (Fried[All Fields] AND ("frailty"[MeSH Terms] OR "frailty"[All Fields]) AND ("abstracting and indexing as topic"[MeSH Terms] OR ("abstracting"[All Fields] AND "indexing"[All Fields] AND "topic"[All Fields]) OR "abstracting and indexing as topic"[All Fields] OR "index"[All Fields]));
12. ("proteins"[MeSH Terms] OR "proteins"[All Fields] OR "protein"[All Fields]) AND (Clinical[All Fields] AND ("frailty"[MeSH Terms] OR "frailty"[All Fields]) AND ("weights and measures"[MeSH Terms] OR ("weights"[All Fields] AND "measures"[All Fields]) OR "weights and measures"[All Fields] OR "scale"[All Fields]));
