# Supplementary material for: Low Protein Intake Is Associated with Frailty in Older Adults: A Systematic Review and Meta-Analysis of Observational Studies
Source: Nutrients. 2018 Sep 19;10(9):1334. doi: 10.3390/nu10091334 (PMC6165078; doi:10.3390/nu10091334)
Supplement: Supplementary file 1 [file nutrients-10-01334-s001.zip › nutrients-339471/Table S2.docx]

**Table S2.** Quality analysis of longitudinal studies.

|  | | | | | | | | | | | | | | | | | | | | | | | |
| --- | --- | --- | --- | --- | --- | --- | --- | --- | --- | --- | --- | --- | --- | --- | --- | --- | --- | --- | --- | --- | --- | --- | --- |
|  | **Item No** | | | | | | | | | | | | | | | | | | | | | |  |
| **Study** | **1** | **2** | **3** | **4** | **5** | **6** | **7** | **8** | **9** | **10** | **11** | **12** | **13** | **14** | **15** | **16** | **17** | **18** | **19** | **20** | **21** | **22** | **Score** |
| Beasley et al., 2010 | X | X | X | X | X | X | X | X | X |  | X | X |  | X | X | X | X | X | X | X | X | X | 20 |
| Shikany et al., 2014 | X | X | X | X | X | X | X | X |  |  | X | X | X | X | X | X | X | X | X | X | X | X | 20 |
| Sandoval et al., 2016 | X | X | X | X | X | X | X | X |  |  | X | X | X | X | X | X | X | X | X | X | X | X | 20 |

**1-** (a) Indicate the study’s design with a commonly used term in the title or the abstract; (b) Provide in the abstract an informative and balanced summary of what was done and what was found; **2-** Explain the scientific background and rationale for the investigation being reported; **3-** State specific objectives, including any prespecified hypotheses; **4-** Present key elements of study design early in the paper; **5-** Describe the setting, locations, and relevant dates, including periods of recruitment, exposure, follow-up, and data collection; **6-** Eligibility criteria, and the sources and methods of selection of participants; **7-** Clearly define all outcomes, exposures, predictors, potential confounders, and effect modifiers. Give diagnostic criteria, if applicable; **8-** For each variable of interest, give sources of data and details of methods of assessment (measurement). Describe comparability of assessment methods if there is more than one group; **9-**Describe any efforts to address potential sources of bias; **10-** Explain how the study size was arrived at; **11-**Explain how quantitative variables were handled in the analyses. If applicable, describe which groupings were chosen and why; **12-**Describe all statistical methods, including those used to control for confounding; **13-** Report numbers of individuals at each stage of study—eg numbers potentially eligible, examined for eligibility, confirmed eligible, included in the study, completing follow-up, and analysed ; **14-** Give characteristics of study participants (eg demographic, clinical, social) and information on exposures and potential confounders; **15-** Report numbers of outcome events or summary measures; **16-** Give unadjusted estimates and, if applicable, confounder-adjusted estimates and their precision (eg, 95% confidence interval). Make clear which confounders were adjusted for and why they were included; **17-**Report other analyses done—eg analyses of subgroups and interactions, and sensitivity analyses; **18-**Summarise key results with reference to study objectives; **19-** Discuss limitations of the study, taking into account sources of potential bias or imprecision. Discuss both direction and magnitude of any potential bias; **20-** Give a cautious overall interpretation of results considering objectives, limitations, multiplicity of analyses, results from similar studies, and other relevant evidence; **21-**Discuss the generalisability (external validity) of the study results; **22-** Give the source of funding and the role of the funders for the present study and, if applicable, for the original study on which the present article is based.
