# Supplementary material for: Low Protein Intake Is Associated with Frailty in Older Adults: A Systematic Review and Meta-Analysis of Observational Studies
Source: Nutrients. 2018 Sep 19;10(9):1334. doi: 10.3390/nu10091334 (PMC6165078; doi:10.3390/nu10091334)
Supplement: Supplementary file 1 [file nutrients-10-01334-s001.zip › nutrients-339471/Table S3.docx]

**Table S3.** Point by point quality analysis.

**Cross-sectional studies**

| **STROBE Statement—checklist of items that should be included in reports of observational studies** | |  |  |  |  |  |
| --- | --- | --- | --- | --- | --- | --- |
|  |  | |  |  |  |  |
| **Article:** Low Nutrient Intake Is an Essential Component of Frailty in Older Persons | **Year:** 2006 | |  |  |  |  |
| **Authors:**  Bartali et al., |  | |  |  |  |  |
|  |  | |  |  |  |  |
|  | **Item No** | | **Recommendation** | **Reported** | **Page (paragraph [line])** | **Note** |
| **Title and abstract** | **1** | | *(a)* Indicate the study’s design with a commonly used term in the title or the abstract | X | Page 1 |  |
|  |  | | *(b)* Provide in the abstract an informative and balanced summary of what was done and what was found | X | Page 1 |  |
| **Introduction** |  | |  |  |  |  |
| *Background/rationale* | **2** | | Explain the scientific background and rationale for the investigation being reported | X | Pages 1-2 |  |
| *Objectives* | **3** | | State specific objectives, including any prespecified hypotheses | X | Page 2 (5) |  |
| **Methods** |  | |  |  |  |  |
| *Study design* | **4** | | Present key elements of study design early in the paper | X | Page 2 (1-4) |  |
| *Setting* | **5** | | Describe the setting, locations, and relevant dates, including periods of recruitment, exposure, follow-up, and data collection | X | Page 2 (1-4) |  |
| *Participants* | **6** | | *(a)* Cohort study—Give the eligibility criteria, and the sources and methods of selection of participants. Describe methods of follow-up |  |  |  |
|  |  | | Case-control study—Give the eligibility criteria, and the sources and methods of case ascertainment and control selection. Give the rationale for the choice of cases and controls | X | Page 2 (2-4) |  |
|  |  | | Cross-sectional study—Give the eligibility criteria, and the sources and methods of selection of participants |  |  |  |
|  |  | | *(b)* Cohort study—For matched studies, give matching criteria and number of exposed and unexposed |  |  |  |
|  |  | | Case-control study—For matched studies, give matching criteria and the number of controls per case |  |  |  |
| *Variables* | **7** | | Clearly define all outcomes, exposures, predictors, potential confounders, and effect modifiers. Give diagnostic criteria, if applicable | X | Pages 2-4 |  |
| *Data sources/ measurement* | **8*** | | For each variable of interest, give sources of data and details of methods of assessment (measurement). Describe comparability of assessment methods if there is more than one group | X | Pages 2-4 |  |
| *Bias* | **9** | | Describe any efforts to address potential sources of bias | X | Page 2 (8) and page 4 | Authors describe that trained researchers were responsible for collecting data |
| *Study size* | **10** | | Explain how the study size was arrived at | X | Page 2 (5) |  |
| *Quantitative variables* | **11** | | Explain how quantitative variables were handled in the analyses. If applicable, describe which groupings were chosen and why | X | Page 4 (2) |  |
| *Statistical methods* | **12** | | *(a)* Describe all statistical methods, including those used to control for confounding | X | Page 4 (2) |  |
|  |  | | *(b)* Describe any methods used to examine subgroups and interactions | X | Page 4 (2) |  |
|  |  | | *(c)* Explain how missing data were addressed | — | — | This information was not mentioned |
|  |  | | *(d)* Cohort study—If applicable, explain how loss to follow-up was addressed |  |  |  |
|  |  | | Case-control study—If applicable, explain how matching of cases and controls was addressed |  |  |  |
|  |  | | Cross-sectional study—If applicable, describe analytical methods taking account of sampling strategy | X | Page 4 (2) |  |
|  |  | | *(e)* Describe any sensitivity analyses | X | Page 4 (2) |  |
| **Results** |  | |  |  |  |  |
| *Participants* | **13*** | | *(a)* Report numbers of individuals at each stage of study—eg numbers potentially eligible, examined for eligibility, confirmed eligible, included in the study, completing follow-up, and analysed | X | Page 2 (6) |  |
|  |  | | *(b)* Give reasons for non-participation at each stage | X | Page 2 (6) |  |
|  |  | | *(c)* Consider use of a flow diagram | — | — |  |
| *Descriptive data* | **14*** | | *(a)* Give characteristics of study participants (eg demographic, clinical, social) and information on exposures and potential confounders | X | Table 1 |  |
|  |  | | *(b)* Indicate number of participants with missing data for each variable of interest | — | — | This information was not mentioned |
|  |  | | *(c)* Cohort study—Summarise follow-up time (eg, average and total amount) |  |  |  |
| *Outcome data* | **15*** | | Cohort study—Report numbers of outcome events or summary measures over time |  |  |  |
|  |  | | Case-control study—Report numbers in each exposure category, or summary measures of exposure |  |  |  |
|  |  | | Cross-sectional study—Report numbers of outcome events or summary measures | X | Tables 2-3 |  |
| *Main results* | **16** | | *(a)* Give unadjusted estimates and, if applicable, confounder-adjusted estimates and their precision (eg, 95% confidence interval). Make clear which confounders were adjusted for and why they were included | X | Tables 2-3 |  |
|  |  | | *(b)* Report category boundaries when continuous variables were categorized | X | Page 4 (2) |  |
|  |  | | *(c)* If relevant, consider translating estimates of relative risk into absolute risk for a meaningful time period |  |  |  |
| *Other analyses* | **17** | | Report other analyses done—eg analyses of subgroups and interactions, and sensitivity analyses | X | Table 3 |  |
| **Discussion** |  | |  |  |  |  |
| *Key results* | **18** | | Summarise key results with reference to study objectives | X | Page 5 |  |
| *Limitations* | **19** | | Discuss limitations of the study, taking into account sources of potential bias or imprecision. Discuss both direction and magnitude of any potential bias | X | Page 5 (5) |  |
| *Interpretation* | **20** | | Give a cautious overall interpretation of results considering objectives, limitations, multiplicity of analyses, results from similar studies, and other relevant evidence | X | Page 5 |  |
| *Generalisability* | **21** | | Discuss the generalisability (external validity) of the study results | X | Page 5 |  |
| **Other information** |  | |  |  |  |  |
| *Funding* | **22** | | Give the source of funding and the role of the funders for the present study and, if applicable, for the original study on which the present article is based | X | Acknowledgements |  |
| **TOTAL SCORE: 22** |  | |  |  |  |  |

| **STROBE Statement—checklist of items that should be included in reports of observational studies** |  |  |  |  |  |
| --- | --- | --- | --- | --- | --- |
|  |  |  |  |  |  |
| **Article:** High protein intake is associated with low prevalence of frailty among old Japanese women: a multicenter cross-sectional study | **Year:** 2013 |  |  |  |  |
| **Authors:**  Kobayashi et al., |  |  |  |  |  |
|  |  |  |  |  |  |
|  | **Item No** | **Recommendation** | **Reported** | **Page (paragraph [line])** | **Note** |
| **Title and abstract** | **1** | *(a)* Indicate the study’s design with a commonly used term in the title or the abstract | X | Page 1 |  |
|  |  | *(b)* Provide in the abstract an informative and balanced summary of what was done and what was found | X | Page 1 |  |
| **Introduction** |  |  |  |  |  |
| *Background/rationale* | **2** | Explain the scientific background and rationale for the investigation being reported | X | Pages 1-2 |  |
| *Objectives* | **3** | State specific objectives, including any prespecified hypotheses | X | Pages 1-2 |  |
| **Methods** |  |  |  |  |  |
| *Study design* | **4** | Present key elements of study design early in the paper | X | Page 2 (3) |  |
| *Setting* | **5** | Describe the setting, locations, and relevant dates, including periods of recruitment, exposure, follow-up, and data collection | X | Page 2 |  |
| *Participants* | **6** | *(a)* Cohort study—Give the eligibility criteria, and the sources and methods of selection of participants. Describe methods of follow-up |  |  |  |
|  |  | Case-control study—Give the eligibility criteria, and the sources and methods of case ascertainment and control selection. Give the rationale for the choice of cases and controls |  |  |  |
|  |  | Cross-sectional study—Give the eligibility criteria, and the sources and methods of selection of participants | X | Page 2 (4) |  |
|  |  | *(b)* Cohort study—For matched studies, give matching criteria and number of exposed and unexposed |  |  |  |
|  |  | Case-control study—For matched studies, give matching criteria and the number of controls per case |  |  |  |
| *Variables* | **7** | Clearly define all outcomes, exposures, predictors, potential confounders, and effect modifiers. Give diagnostic criteria, if applicable | X | Pages 2-3 |  |
| *Data sources/ measurement* | **8*** | For each variable of interest, give sources of data and details of methods of assessment (measurement). Describe comparability of assessment methods if there is more than one group | X | Pages 2-3 |  |
| *Bias* | **9** | Describe any efforts to address potential sources of bias | — | — | This information was not added |
| *Study size* | **10** | Explain how the study size was arrived at | X | — | The authors did not describe how the study size arrived at. However, it was clear that sample size was defined by convenience |
| *Quantitative variables* | **11** | Explain how quantitative variables were handled in the analyses. If applicable, describe which groupings were chosen and why | X |  |  |
| *Statistical methods* | **12** | *(a)* Describe all statistical methods, including those used to control for confounding | X | Pages 2-3 |  |
|  |  | *(b)* Describe any methods used to examine subgroups and interactions | X | Page 3 |  |
|  |  | *(c)* Explain how missing data were addressed | X | Page 2 | Individuals with missing data were excluded |
|  |  | *(d)* Cohort study—If applicable, explain how loss to follow-up was addressed |  |  |  |
|  |  | Case-control study—If applicable, explain how matching of cases and controls was addressed |  |  |  |
|  |  | Cross-sectional study—If applicable, describe analytical methods taking account of sampling strategy |  |  |  |
|  |  | *(e)* Describe any sensitivity analyses | X | Pages 2-3 |  |
| **Results** |  |  |  |  |  |
| *Participants* | **13*** | *(a)* Report numbers of individuals at each stage of study—eg numbers potentially eligible, examined for eligibility, confirmed eligible, included in the study, completing follow-up, and analysed | X | Page 2 |  |
|  |  | *(b)* Give reasons for non-participation at each stage | X | Page 2 |  |
|  |  | *(c)* Consider use of a flow diagram | — | — |  |
| *Descriptive data* | **14*** | *(a)* Give characteristics of study participants (eg demographic, clinical, social) and information on exposures and potential confounders | X | Table 1 |  |
|  |  | *(b)* Indicate number of participants with missing data for each variable of interest | — | — | Individuals with missing data were excluded |
|  |  | *(c)* Cohort study—Summarise follow-up time (eg, average and total amount) |  |  |  |
| *Outcome data* | **15*** | Cohort study—Report numbers of outcome events or summary measures over time |  |  |  |
|  |  | Case-control study—Report numbers in each exposure category, or summary measures of exposure |  |  |  |
|  |  | Cross-sectional study—Report numbers of outcome events or summary measures | X | Tables 2-3 |  |
| *Main results* | **16** | *(a)* Give unadjusted estimates and, if applicable, confounder-adjusted estimates and their precision (eg, 95% confidence interval). Make clear which confounders were adjusted for and why they were included | X | Table 3 |  |
|  |  | *(b)* Report category boundaries when continuous variables were categorized | X | Table 3 |  |
|  |  | *(c)* If relevant, consider translating estimates of relative risk into absolute risk for a meaningful time period |  |  |  |
| *Other analyses* | **17** | Report other analyses done—eg analyses of subgroups and interactions, and sensitivity analyses | X | Table 3 |  |
| **Discussion** |  |  |  |  |  |
| *Key results* | **18** | Summarise key results with reference to study objectives | X | Pages 6, 8-9 |  |
| *Limitations* | **19** | Discuss limitations of the study, taking into account sources of potential bias or imprecision. Discuss both direction and magnitude of any potential bias | X | Pages 8-9 |  |
| *Interpretation* | **20** | Give a cautious overall interpretation of results considering objectives, limitations, multiplicity of analyses, results from similar studies, and other relevant evidence | X | Pages 6, 8-9 |  |
| *Generalisability* | **21** | Discuss the generalisability (external validity) of the study results | X | Pages 6, 8-9 |  |
| **Other information** |  |  |  |  |  |
| *Funding* | **22** | Give the source of funding and the role of the funders for the present study and, if applicable, for the original study on which the present article is based | — | — |  |
| **TOTAL SCORE: 20** |  |  |  |  |  |
| **STROBE Statement—checklist of items that should be included in reports of observational studies** |  |  |  |  |  |
|  |  |  |  |  |  |
| **Article: Distribution but not amount of protein intake is associated with frailty: a cross-sectional investigation in the region of Nürnbergdy** | **Year: 2013** |  |  |  |  |
| **Authors: Bollwein et al.,** |  |  |  |  |  |
|  |  |  |  |  |  |
|  | **Item No** | Recommendation | Reported | Page (paragraph [line]) | Note |
| **Title and abstract** | **1** | (a) Indicate the study’s design with a commonly used term in the title or the abstract | X | Page 1 |  |
|  |  | (b) Provide in the abstract an informative and balanced summary of what was done and what was found | X | Page 1 |  |
| **Introduction** |  |  |  |  |  |
| **Background/rationale** | **2** | Explain the scientific background and rationale for the investigation being reported | X | Pages 1-2 |  |
| **Objectives** | **3** | State specific objectives, including any prespecified hypotheses | X | Pages 1-2 |  |
| **Methods** |  |  |  |  |  |
| **Study design** | **4** | Present key elements of study design early in the paper | X | Page 2 (4) |  |
| **Setting** | **5** | Describe the setting, locations, and relevant dates, including periods of recruitment, exposure, follow-up, and data collection | X | Page 2 (4) |  |
| **Participants** | **6** | (a) Cohort study—Give the eligibility criteria, and the sources and methods of selection of participants. Describe methods of follow-up |  |  |  |
|  |  | Case-control study—Give the eligibility criteria, and the sources and methods of case ascertainment and control selection. Give the rationale for the choice of cases and controls |  |  |  |
|  |  | Cross-sectional study—Give the eligibility criteria, and the sources and methods of selection of participants | X | Page 2 (4) |  |
|  |  | (b) Cohort study—For matched studies, give matching criteria and number of exposed and unexposed |  |  |  |
|  |  | Case-control study—For matched studies, give matching criteria and the number of controls per case |  |  |  |
| **Variables** | **7** | Clearly define all outcomes, exposures, predictors, potential confounders, and effect modifiers. Give diagnostic criteria, if applicable | X | Pages 2-3 |  |
| **Data sources/ measurement** | **8*** | For each variable of interest, give sources of data and details of methods of assessment (measurement). Describe comparability of assessment methods if there is more than one group | X | Pages 2-4 |  |
| **Bias** | **9** | Describe any efforts to address potential sources of bias | — | — |  |
| **Study size** | **10** | Explain how the study size was arrived at | — | — |  |
| **Quantitative variables** | **11** | Explain how quantitative variables were handled in the analyses. If applicable, describe which groupings were chosen and why | X | Pages 2-4 |  |
| **Statistical methods** | **12** | (a) Describe all statistical methods, including those used to control for confounding | X | Pages 3-4 |  |
|  |  | (b) Describe any methods used to examine subgroups and interactions | X | Pages 3-4 |  |
|  |  | (c) Explain how missing data were addressed | X | Page 4 (2) | Volunteers with missing data were excluded |
|  |  | (d) Cohort study—If applicable, explain how loss to follow-up was addressed |  |  |  |
|  |  | Case-control study—If applicable, explain how matching of cases and controls was addressed |  |  |  |
|  |  | Cross-sectional study—If applicable, describe analytical methods taking account of sampling strategy |  |  |  |
|  |  | (e) Describe any sensitivity analyses |  |  |  |
| **Results** |  |  |  |  |  |
| **Participants** | **13*** | (a) Report numbers of individuals at each stage of study—eg numbers potentially eligible, examined for eligibility, confirmed eligible, included in the study, completing follow-up, and analysed | — | — | This informantion was not added |
|  |  | (b) Give reasons for non-participation at each stage | — | — | This informantion was not added |
|  |  | (c) Consider use of a flow diagram | — | — | This informantion was not added |
| **Descriptive data** | **14*** | (a) Give characteristics of study participants (eg demographic, clinical, social) and information on exposures and potential confounders | X | Table 1 |  |
|  |  | (b) Indicate number of participants with missing data for each variable of interest | — | — | These volunteers were excluded |
|  |  | (c) Cohort study—Summarise follow-up time (eg, average and total amount) |  |  |  |
| **Outcome data** | **15*** | Cohort study—Report numbers of outcome events or summary measures over time |  |  |  |
|  |  | Case-control study—Report numbers in each exposure category, or summary measures of exposure |  |  |  |
|  |  | Cross-sectional study—Report numbers of outcome events or summary measures | X | Tables 2-5 and Figure 1 |  |
| **Main results** | **16** | (a) Give unadjusted estimates and, if applicable, confounder-adjusted estimates and their precision (eg, 95% confidence interval). Make clear which confounders were adjusted for and why they were included | X | Tables 3-5 |  |
|  |  | (b) Report category boundaries when continuous variables were categorized |  | Pages 2-3 |  |
|  |  | (c) If relevant, consider translating estimates of relative risk into absolute risk for a meaningful time period |  |  |  |
| **Other analyses** | **17** | Report other analyses done—eg analyses of subgroups and interactions, and sensitivity analyses | X | Figure 1 |  |
| **Discussion** |  |  |  |  |  |
| **Key results** | **18** | Summarise key results with reference to study objectives | X | Pages 4-6 |  |
| **Limitations** | **19** | Discuss limitations of the study, taking into account sources of potential bias or imprecision. Discuss both direction and magnitude of any potential bias | X | Pages 5-6 |  |
| **Interpretation** | **20** | Give a cautious overall interpretation of results considering objectives, limitations, multiplicity of analyses, results from similar studies, and other relevant evidence | X | Pages 4-6 |  |
| **Generalisability** | **21** | Discuss the generalisability (external validity) of the study results | X | Pages 4-6 |  |
| **Other information** |  |  |  |  |  |
| **Funding** | **22** | Give the source of funding and the role of the funders for the present study and, if applicable, for the original study on which the present article is based | X | Acknowledgements |  |
| **TOTAL SCORE: 19** |  |  |  |  |  |

| **STROBE Statement—checklist of items that should be included in reports of observational studies** |  |  |  |  |  |
| --- | --- | --- | --- | --- | --- |
|  |  |  |  |  |  |
| **Article:** Macronutrients, Diet Quality, and Frailty in Older Men | **Year:** 2014 |  |  |  |  |
| **Authors:**  Shikany et a., |  |  |  |  |  |
|  |  |  |  |  |  |
|  | **Item No** | **Recommendation** | **Reported** | **Page (paragraph [line])** | **Note** |
| **Title and abstract** | **1** | *(a)* Indicate the study’s design with a commonly used term in the title or the abstract | X | Page 695 |  |
|  |  | *(b)* Provide in the abstract an informative and balanced summary of what was done and what was found | X | Page 695 |  |
| **Introduction** |  |  |  |  |  |
| *Background/rationale* | **2** | Explain the scientific background and rationale for the investigation being reported | X | Pages 695-696 |  |
| *Objectives* | **3** | State specific objectives, including any prespecified hypotheses | X | Pages 695-696 |  |
| **Methods** |  |  |  |  |  |
| *Study design* | **4** | Present key elements of study design early in the paper | X | Page 696 |  |
| *Setting* | **5** | Describe the setting, locations, and relevant dates, including periods of recruitment, exposure, follow-up, and data collection | X | Page 696 |  |
| *Participants* | **6** | *(a)* Cohort study—Give the eligibility criteria, and the sources and methods of selection of participants. Describe methods of follow-up |  |  |  |
|  |  | Case-control study—Give the eligibility criteria, and the sources and methods of case ascertainment and control selection. Give the rationale for the choice of cases and controls |  |  |  |
|  |  | Cross-sectional study—Give the eligibility criteria, and the sources and methods of selection of participants | X | Pages 696-697 (2) |  |
|  |  | *(b)* Cohort study—For matched studies, give matching criteria and number of exposed and unexposed |  |  |  |
|  |  | Case-control study—For matched studies, give matching criteria and the number of controls per case |  |  |  |
| *Variables* | **7** | Clearly define all outcomes, exposures, predictors, potential confounders, and effect modifiers. Give diagnostic criteria, if applicable | X | Pages 696-697 |  |
| *Data sources/ measurement* | **8*** | For each variable of interest, give sources of data and details of methods of assessment (measurement). Describe comparability of assessment methods if there is more than one group | X | Pages 696-697 |  |
| *Bias* | **9** | Describe any efforts to address potential sources of bias | — | — | This information was not mentioned |
| *Study size* | **10** | Explain how the study size was arrived at | — | — | This information was not mentioned |
| *Quantitative variables* | **11** | Explain how quantitative variables were handled in the analyses. If applicable, describe which groupings were chosen and why | X | Pages 696-697 |  |
| *Statistical methods* | **12** | *(a)* Describe all statistical methods, including those used to control for confounding | X | Page 697 |  |
|  |  | *(b)* Describe any methods used to examine subgroups and interactions | X | Page 697 |  |
|  |  | *(c)* Explain how missing data were addressed | X | Page 697 |  |
|  |  | *(d)* Cohort study—If applicable, explain how loss to follow-up was addressed |  |  |  |
|  |  | Case-control study—If applicable, explain how matching of cases and controls was addressed |  |  |  |
|  |  | Cross-sectional study—If applicable, describe analytical methods taking account of sampling strategy | X | Page 697 |  |
|  |  | *(e)* Describe any sensitivity analyses |  |  |  |
| **Results** |  |  |  |  |  |
| *Participants* | **13*** | *(a)* Report numbers of individuals at each stage of study—eg numbers potentially eligible, examined for eligibility, confirmed eligible, included in the study, completing follow-up, and analysed | X | Page 697 |  |
|  |  | *(b)* Give reasons for non-participation at each stage | X | Page 697 |  |
|  |  | *(c)* Consider use of a flow diagram | — | — | This information was not mentioned |
| *Descriptive data* | **14*** | *(a)* Give characteristics of study participants (eg demographic, clinical, social) and information on exposures and potential confounders | X | Table 1 |  |
|  |  | *(b)* Indicate number of participants with missing data for each variable of interest | — | — | This information was not mentioned |
|  |  | *(c)* Cohort study—Summarise follow-up time (eg, average and total amount) |  |  |  |
| *Outcome data* | **15*** | Cohort study—Report numbers of outcome events or summary measures over time |  |  |  |
|  |  | Case-control study—Report numbers in each exposure category, or summary measures of exposure |  |  |  |
|  |  | Cross-sectional study—Report numbers of outcome events or summary measures | X | Table 2-3 |  |
| *Main results* | **16** | *(a)* Give unadjusted estimates and, if applicable, confounder-adjusted estimates and their precision (eg, 95% confidence interval). Make clear which confounders were adjusted for and why they were included | X | Table 2-3 |  |
|  |  | *(b)* Report category boundaries when continuous variables were categorized | X | Page 697 |  |
|  |  | *(c)* If relevant, consider translating estimates of relative risk into absolute risk for a meaningful time period |  |  |  |
| *Other analyses* | **17** | Report other analyses done—eg analyses of subgroups and interactions, and sensitivity analyses | X | Tables 2-3 |  |
| **Discussion** |  |  |  |  |  |
| *Key results* | **18** | Summarise key results with reference to study objectives | X | Pages 698-701 |  |
| *Limitations* | **19** | Discuss limitations of the study, taking into account sources of potential bias or imprecision. Discuss both direction and magnitude of any potential bias | X | Pages 698-701 |  |
| *Interpretation* | **20** | Give a cautious overall interpretation of results considering objectives, limitations, multiplicity of analyses, results from similar studies, and other relevant evidence | X | Pages 698-701 |  |
| *Generalisability* | **21** | Discuss the generalisability (external validity) of the study results | X | Pages 698-701 |  |
| **Other information** |  |  |  |  |  |
| *Funding* | **22** | Give the source of funding and the role of the funders for the present study and, if applicable, for the original study on which the present article is based | X | Funding |  |
| **TOTAL SCORE: 20** |  |  |  |  |  |

| **STROBE Statement—checklist of items that should be included in reports of observational studies** |  |  |  |  |  |
| --- | --- | --- | --- | --- | --- |
|  |  |  |  |  |  |
| **Article:** | **Year:** |  |  |  |  |
| **Authors:** |  |  |  |  |  |
|  |  |  |  |  |  |
|  | **Item No** | **Recommendation** | **Reported** | **Page (paragraph [line])** | **Note** |
| **Title and abstract** | **1** | *(a)* Indicate the study’s design with a commonly used term in the title or the abstract | X | 672.e7 |  |
|  |  | *(b)* Provide in the abstract an informative and balanced summary of what was done and what was found | X | 672.e7 |  |
| **Introduction** |  |  |  |  |  |
| *Background/rationale* | **2** | Explain the scientific background and rationale for the investigation being reported | X | 672.e7-672.e8 |  |
| *Objectives* | **3** | State specific objectives, including any prespecified hypotheses | X | 672.e7-672.e8 |  |
| **Methods** |  |  |  |  |  |
| *Study design* | **4** | Present key elements of study design early in the paper | X | 672.e8 |  |
| *Setting* | **5** | Describe the setting, locations, and relevant dates, including periods of recruitment, exposure, follow-up, and data collection | X | 672.e8 |  |
| *Participants* | **6** | *(a)* Cohort study—Give the eligibility criteria, and the sources and methods of selection of participants. Describe methods of follow-up |  |  |  |
|  |  | Case-control study—Give the eligibility criteria, and the sources and methods of case ascertainment and control selection. Give the rationale for the choice of cases and controls |  |  |  |
|  |  | Cross-sectional study—Give the eligibility criteria, and the sources and methods of selection of participants | X | 672.e8 | The authors mentioned the source of data |
|  |  | *(b)* Cohort study—For matched studies, give matching criteria and number of exposed and unexposed |  |  |  |
|  |  | Case-control study—For matched studies, give matching criteria and the number of controls per case |  |  |  |
| *Variables* | **7** | Clearly define all outcomes, exposures, predictors, potential confounders, and effect modifiers. Give diagnostic criteria, if applicable | X | 672.e8 |  |
| *Data sources/ measurement* | **8*** | For each variable of interest, give sources of data and details of methods of assessment (measurement). Describe comparability of assessment methods if there is more than one group | X | 672.e8 |  |
| *Bias* | **9** | Describe any efforts to address potential sources of bias | — | — | This information was not mentioned |
| *Study size* | **10** | Explain how the study size was arrived at | — | — | This information was not mentioned |
| *Quantitative variables* | **11** | Explain how quantitative variables were handled in the analyses. If applicable, describe which groupings were chosen and why | X | 672.e8 |  |
| *Statistical methods* | **12** | *(a)* Describe all statistical methods, including those used to control for confounding | X | 672.e8 |  |
|  |  | *(b)* Describe any methods used to examine subgroups and interactions | X | 672.e8 |  |
|  |  | *(c)* Explain how missing data were addressed | X | 672.e9 (1) |  |
|  |  | *(d)* Cohort study—If applicable, explain how loss to follow-up was addressed |  |  |  |
|  |  | Case-control study—If applicable, explain how matching of cases and controls was addressed |  |  |  |
|  |  | Cross-sectional study—If applicable, describe analytical methods taking account of sampling strategy | X | 672.e8 |  |
|  |  | *(e)* Describe any sensitivity analyses | X | 672.e8 |  |
| **Results** |  |  |  |  |  |
| *Participants* | **13*** | *(a)* Report numbers of individuals at each stage of study—eg numbers potentially eligible, examined for eligibility, confirmed eligible, included in the study, completing follow-up, and analysed | X | 672.e9 (1) |  |
|  |  | *(b)* Give reasons for non-participation at each stage | X | 672.e9 (1) |  |
|  |  | *(c)* Consider use of a flow diagram | — | — | This information was not mentioned |
| *Descriptive data* | **14*** | *(a)* Give characteristics of study participants (eg demographic, clinical, social) and information on exposures and potential confounders | X | Table 1 |  |
|  |  | *(b)* Indicate number of participants with missing data for each variable of interest | X |  |  |
|  |  | *(c)* Cohort study—Summarise follow-up time (eg, average and total amount) |  |  |  |
| *Outcome data* | **15*** | Cohort study—Report numbers of outcome events or summary measures over time |  |  |  |
|  |  | Case-control study—Report numbers in each exposure category, or summary measures of exposure |  |  |  |
|  |  | Cross-sectional study—Report numbers of outcome events or summary measures | X | Table 2-3 and Supplementary material |  |
| *Main results* | **16** | *(a)* Give unadjusted estimates and, if applicable, confounder-adjusted estimates and their precision (eg, 95% confidence interval). Make clear which confounders were adjusted for and why they were included | X | Tables 2-3 |  |
|  |  | *(b)* Report category boundaries when continuous variables were categorized | X | 672.e8 |  |
|  |  | *(c)* If relevant, consider translating estimates of relative risk into absolute risk for a meaningful time period |  |  |  |
| *Other analyses* | **17** | Report other analyses done—eg analyses of subgroups and interactions, and sensitivity analyses | X | Supplementary material |  |
| **Discussion** |  |  |  |  |  |
| *Key results* | **18** | Summarise key results with reference to study objectives | X | 672.e10 |  |
| *Limitations* | **19** | Discuss limitations of the study, taking into account sources of potential bias or imprecision. Discuss both direction and magnitude of any potential bias | X | 672.e10 |  |
| *Interpretation* | **20** | Give a cautious overall interpretation of results considering objectives, limitations, multiplicity of analyses, results from similar studies, and other relevant evidence | X | 672.e10 |  |
| *Generalisability* | **21** | Discuss the generalisability (external validity) of the study results | X | 672.e10 |  |
| **Other information** |  |  |  |  |  |
| *Funding* | **22** | Give the source of funding and the role of the funders for the present study and, if applicable, for the original study on which the present article is based | X | Acknowledgments |  |
| **TOTAL SCORE: 20** |  |  |  |  |  |

| **STROBE Statement—checklist of items that should be included in reports of observational studies** |  |  |  |  |  |
| --- | --- | --- | --- | --- | --- |
|  |  |  |  |  |  |
| **Article:** Sex Difference in the Association Between Protein Intake and Frailty: Assessed Using the Kihon Checklist Indexes Among Older Adults | **Year:** 2018 |  |  |  |  |
| **Authors:**  Nanri et al., 2016 |  |  |  |  |  |
|  |  |  |  |  |  |
|  | **Item No** | **Recommendation** | **Reported** | **Page (paragraph [line])** | **Note** |
| **Title and abstract** | **1** | *(a)* Indicate the study’s design with a commonly used term in the title or the abstract | X | Page 1 |  |
|  |  | *(b)* Provide in the abstract an informative and balanced summary of what was done and what was found | X | Page 1 |  |
| **Introduction** |  |  |  |  |  |
| *Background/rationale* | **2** | Explain the scientific background and rationale for the investigation being reported | X | Pages 1-2 |  |
| *Objectives* | **3** | State specific objectives, including any prespecified hypotheses | X | Page 2 |  |
| **Methods** |  |  |  |  |  |
| *Study design* | **4** | Present key elements of study design early in the paper | X | Page 2 |  |
| *Setting* | **5** | Describe the setting, locations, and relevant dates, including periods of recruitment, exposure, follow-up, and data collection | X | Page 2 |  |
| *Participants* | **6** | *(a)* Cohort study—Give the eligibility criteria, and the sources and methods of selection of participants. Describe methods of follow-up |  |  |  |
|  |  | Case-control study—Give the eligibility criteria, and the sources and methods of case ascertainment and control selection. Give the rationale for the choice of cases and controls |  |  |  |
|  |  | Cross-sectional study—Give the eligibility criteria, and the sources and methods of selection of participants | X | Page 2 |  |
|  |  | *(b)* Cohort study—For matched studies, give matching criteria and number of exposed and unexposed |  |  |  |
|  |  | Case-control study—For matched studies, give matching criteria and the number of controls per case |  |  |  |
| *Variables* | **7** | Clearly define all outcomes, exposures, predictors, potential confounders, and effect modifiers. Give diagnostic criteria, if applicable | X | Pages '2-3 |  |
| *Data sources/ measurement* | **8*** | For each variable of interest, give sources of data and details of methods of assessment (measurement). Describe comparability of assessment methods if there is more than one group | X | Pages '2-3 |  |
| *Bias* | **9** | Describe any efforts to address potential sources of bias | — | — | This information was not mentioned |
| *Study size* | **10** | Explain how the study size was arrived at | — | — | This information was not mentioned |
| *Quantitative variables* | **11** | Explain how quantitative variables were handled in the analyses. If applicable, describe which groupings were chosen and why | X | Pages '2-3 |  |
| *Statistical methods* | **12** | *(a)* Describe all statistical methods, including those used to control for confounding | X | Page 3 |  |
|  |  | *(b)* Describe any methods used to examine subgroups and interactions | X | Page 3 |  |
|  |  | *(c)* Explain how missing data were addressed | X | Page 2 | Volunteers with missing data were excluded |
|  |  | *(d)* Cohort study—If applicable, explain how loss to follow-up was addressed |  |  |  |
|  |  | Case-control study—If applicable, explain how matching of cases and controls was addressed |  |  |  |
|  |  | Cross-sectional study—If applicable, describe analytical methods taking account of sampling strategy |  |  |  |
|  |  | *(e)* Describe any sensitivity analyses |  |  |  |
| **Results** |  |  |  |  |  |
| *Participants* | **13*** | *(a)* Report numbers of individuals at each stage of study—eg numbers potentially eligible, examined for eligibility, confirmed eligible, included in the study, completing follow-up, and analysed | X | Page 2 |  |
|  |  | *(b)* Give reasons for non-participation at each stage | X | Page 2 |  |
|  |  | *(c)* Consider use of a flow diagram | — | — |  |
| *Descriptive data* | **14*** | *(a)* Give characteristics of study participants (eg demographic, clinical, social) and information on exposures and potential confounders | X | Table 1 |  |
|  |  | *(b)* Indicate number of participants with missing data for each variable of interest | X | Page 2 | Volunteers with missing data were excluded |
|  |  | *(c)* Cohort study—Summarise follow-up time (eg, average and total amount) |  |  |  |
| *Outcome data* | **15*** | Cohort study—Report numbers of outcome events or summary measures over time |  |  |  |
|  |  | Case-control study—Report numbers in each exposure category, or summary measures of exposure |  |  |  |
|  |  | Cross-sectional study—Report numbers of outcome events or summary measures | X | Table 2 and Supplemental material |  |
| *Main results* | **16** | *(a)* Give unadjusted estimates and, if applicable, confounder-adjusted estimates and their precision (eg, 95% confidence interval). Make clear which confounders were adjusted for and why they were included | X | Table 2 and Supplemental material |  |
|  |  | *(b)* Report category boundaries when continuous variables were categorized | X | Table 2 and Supplemental material |  |
|  |  | *(c)* If relevant, consider translating estimates of relative risk into absolute risk for a meaningful time period |  |  |  |
| *Other analyses* | **17** | Report other analyses done—eg analyses of subgroups and interactions, and sensitivity analyses | X | Supplemental material |  |
| **Discussion** |  |  |  |  |  |
| *Key results* | **18** | Summarise key results with reference to study objectives | X |  |  |
| *Limitations* | **19** | Discuss limitations of the study, taking into account sources of potential bias or imprecision. Discuss both direction and magnitude of any potential bias | X | Page 4 |  |
| *Interpretation* | **20** | Give a cautious overall interpretation of results considering objectives, limitations, multiplicity of analyses, results from similar studies, and other relevant evidence | X | Page 4 |  |
| *Generalisability* | **21** | Discuss the generalisability (external validity) of the study results | X | Page 4 |  |
| **Other information** |  |  |  |  |  |
| *Funding* | **22** | Give the source of funding and the role of the funders for the present study and, if applicable, for the original study on which the present article is based | X | Acknowledgments |  |
| **TOTAL SCORE: 20** |  |  |  |  |  |

| **STROBE Statement—checklist of items that should be included in reports of observational studies** |  |  |  |  |  |
| --- | --- | --- | --- | --- | --- |
|  |  |  |  |  |  |
| **Article:** Diet with a combination of high protein and high total antioxidant capacity is strongly associated with low prevalence of frailty among old Japanese women: a multicenter cross-sectional studyY | **Year:** 2017 |  |  |  |  |
| **Authors:**  Kobayashi et al., |  |  |  |  |  |
|  |  |  |  |  |  |
|  | **Item No** | **Recommendation** | **Reported** | **Page (paragraph [line])** | **Note** |
| **Title and abstract** | **1** | *(a)* Indicate the study’s design with a commonly used term in the title or the abstract | X | Page 1 |  |
|  |  | *(b)* Provide in the abstract an informative and balanced summary of what was done and what was found | X | Page 1 |  |
| **Introduction** |  |  |  |  |  |
| *Background/rationale* | **2** | Explain the scientific background and rationale for the investigation being reported | X | Page 2 |  |
| *Objectives* | **3** | State specific objectives, including any prespecified hypotheses | X | Page 2 |  |
| **Methods** |  |  |  |  |  |
| *Study design* | **4** | Present key elements of study design early in the paper | X | Page 2 |  |
| *Setting* | **5** | Describe the setting, locations, and relevant dates, including periods of recruitment, exposure, follow-up, and data collection | X | Page 2 |  |
| *Participants* | **6** | *(a)* Cohort study—Give the eligibility criteria, and the sources and methods of selection of participants. Describe methods of follow-up |  |  |  |
|  |  | Case-control study—Give the eligibility criteria, and the sources and methods of case ascertainment and control selection. Give the rationale for the choice of cases and controls |  |  |  |
|  |  | Cross-sectional study—Give the eligibility criteria, and the sources and methods of selection of participants | X | Page 2 |  |
|  |  | *(b)* Cohort study—For matched studies, give matching criteria and number of exposed and unexposed |  |  |  |
|  |  | Case-control study—For matched studies, give matching criteria and the number of controls per case |  |  |  |
| *Variables* | **7** | Clearly define all outcomes, exposures, predictors, potential confounders, and effect modifiers. Give diagnostic criteria, if applicable | X | Pages 2-3 |  |
| *Data sources/ measurement* | **8*** | For each variable of interest, give sources of data and details of methods of assessment (measurement). Describe comparability of assessment methods if there is more than one group | X | Pages 2-3 |  |
| *Bias* | **9** | Describe any efforts to address potential sources of bias | — | — | This information was not mentioned |
| *Study size* | **10** | Explain how the study size was arrived at | — | — | This information was not mentioned |
| *Quantitative variables* | **11** | Explain how quantitative variables were handled in the analyses. If applicable, describe which groupings were chosen and why | X | Pages 2-3 |  |
| *Statistical methods* | **12** | *(a)* Describe all statistical methods, including those used to control for confounding | X | Pages 3-4 |  |
|  |  | *(b)* Describe any methods used to examine subgroups and interactions | X | Pages 3-4 |  |
|  |  | *(c)* Explain how missing data were addressed | X | Page 4 | Individuals with missing data were excluded |
|  |  | *(d)* Cohort study—If applicable, explain how loss to follow-up was addressed |  |  |  |
|  |  | Case-control study—If applicable, explain how matching of cases and controls was addressed |  |  |  |
|  |  | Cross-sectional study—If applicable, describe analytical methods taking account of sampling strategy |  |  |  |
|  |  | *(e)* Describe any sensitivity analyses | X | Pages 3-4 |  |
| **Results** |  |  |  |  |  |
| *Participants* | **13*** | *(a)* Report numbers of individuals at each stage of study—eg numbers potentially eligible, examined for eligibility, confirmed eligible, included in the study, completing follow-up, and analysed | X | Page 4 |  |
|  |  | *(b)* Give reasons for non-participation at each stage | X | Page 4 |  |
|  |  | *(c)* Consider use of a flow diagram | — | — | This information was not mentioned |
| *Descriptive data* | **14*** | *(a)* Give characteristics of study participants (eg demographic, clinical, social) and information on exposures and potential confounders | X | Table 1 |  |
|  |  | *(b)* Indicate number of participants with missing data for each variable of interest | X | Page 4 | Individuals with missing data were excluded |
|  |  | *(c)* Cohort study—Summarise follow-up time (eg, average and total amount) |  |  |  |
| *Outcome data* | **15*** | Cohort study—Report numbers of outcome events or summary measures over time |  |  |  |
|  |  | Case-control study—Report numbers in each exposure category, or summary measures of exposure |  |  |  |
|  |  | Cross-sectional study—Report numbers of outcome events or summary measures | X | Tables 2-5 |  |
| *Main results* | **16** | *(a)* Give unadjusted estimates and, if applicable, confounder-adjusted estimates and their precision (eg, 95% confidence interval). Make clear which confounders were adjusted for and why they were included | X | Tables 2-5 |  |
|  |  | *(b)* Report category boundaries when continuous variables were categorized | X | Tables 2-5 |  |
|  |  | *(c)* If relevant, consider translating estimates of relative risk into absolute risk for a meaningful time period |  |  |  |
| *Other analyses* | **17** | Report other analyses done—eg analyses of subgroups and interactions, and sensitivity analyses | X | Tables 2-5 |  |
| **Discussion** |  |  |  |  |  |
| *Key results* | **18** | Summarise key results with reference to study objectives | X | Pages 6-9 and page 10 |  |
| *Limitations* | **19** | Discuss limitations of the study, taking into account sources of potential bias or imprecision. Discuss both direction and magnitude of any potential bias | X | Pages 6-9 and page 10 |  |
| *Interpretation* | **20** | Give a cautious overall interpretation of results considering objectives, limitations, multiplicity of analyses, results from similar studies, and other relevant evidence | X | Pages 6-9 and page 10 |  |
| *Generalisability* | **21** | Discuss the generalisability (external validity) of the study results | X | Pages 6-9 and page 10 |  |
| **Other information** |  |  |  |  |  |
| *Funding* | **22** | Give the source of funding and the role of the funders for the present study and, if applicable, for the original study on which the present article is based | X | Funding |  |
| **TOTAL SCORE: 20** |  |  |  |  |  |

**Longitudinal studies**

| **STROBE Statement—checklist of items that should be included in reports of observational studies** |  |  |  |  |  |
| --- | --- | --- | --- | --- | --- |
|  |  |  |  |  |  |
| **Article:** | **Year:** |  |  |  |  |
| **Authors:** |  |  |  |  |  |
|  |  |  |  |  |  |
|  | **Item No** | **Recommendation** | **Reported** | **Page (paragraph [line])** | **Note** |
| **Title and abstract** | **1** | *(a)* Indicate the study’s design with a commonly used term in the title or the abstract | X | Page 1 |  |
|  |  | *(b)* Provide in the abstract an informative and balanced summary of what was done and what was found | X | Pages 1-2 |  |
| **Introduction** |  |  |  |  |  |
| *Background/rationale* | **2** | Explain the scientific background and rationale for the investigation being reported | X | Page 2 |  |
| *Objectives* | **3** | State specific objectives, including any prespecified hypotheses | X | Page 2 |  |
| **Methods** |  |  |  |  |  |
| *Study design* | **4** | Present key elements of study design early in the paper | X | Pages 2-3 |  |
| *Setting* | **5** | Describe the setting, locations, and relevant dates, including periods of recruitment, exposure, follow-up, and data collection | X | Pages 2-3 |  |
| *Participants* | **6** | *(a)* Cohort study—Give the eligibility criteria, and the sources and methods of selection of participants. Describe methods of follow-up | X | Pages 2-3 |  |
|  |  | Case-control study—Give the eligibility criteria, and the sources and methods of case ascertainment and control selection. Give the rationale for the choice of cases and controls |  |  |  |
|  |  | Cross-sectional study—Give the eligibility criteria, and the sources and methods of selection of participants |  |  |  |
|  |  | *(b)* Cohort study—For matched studies, give matching criteria and number of exposed and unexposed |  |  |  |
|  |  | Case-control study—For matched studies, give matching criteria and the number of controls per case |  |  |  |
| *Variables* | **7** | Clearly define all outcomes, exposures, predictors, potential confounders, and effect modifiers. Give diagnostic criteria, if applicable | X | Pages 2-4 |  |
| *Data sources/ measurement* | **8*** | For each variable of interest, give sources of data and details of methods of assessment (measurement). Describe comparability of assessment methods if there is more than one group | X | Pages 2-4 |  |
| *Bias* | **9** | Describe any efforts to address potential sources of bias | X | Pages 3-4 | Researchers applied a calibrated protein estimation model to reduce bias associated with FFQ evaluation |
| *Study size* | **10** | Explain how the study size was arrived at | — | — | This information was not mentioned |
| *Quantitative variables* | **11** | Explain how quantitative variables were handled in the analyses. If applicable, describe which groupings were chosen and why | X | Pages 2-4 |  |
| *Statistical methods* | **12** | *(a)* Describe all statistical methods, including those used to control for confounding | X | Pages 4-5 |  |
|  |  | *(b)* Describe any methods used to examine subgroups and interactions | X | Pages 4-5 |  |
|  |  | *(c)* Explain how missing data were addressed | X | Page 3 |  |
|  |  | *(d)* Cohort study—If applicable, explain how loss to follow-up was addressed | X | Page 3 |  |
|  |  | Case-control study—If applicable, explain how matching of cases and controls was addressed |  |  |  |
|  |  | Cross-sectional study—If applicable, describe analytical methods taking account of sampling strategy |  |  |  |
|  |  | *(e)* Describe any sensitivity analyses |  |  |  |
| **Results** |  |  |  |  |  |
| *Participants* | **13*** | *(a)* Report numbers of individuals at each stage of study—eg numbers potentially eligible, examined for eligibility, confirmed eligible, included in the study, completing follow-up, and analysed | — | — | — |
|  |  | *(b)* Give reasons for non-participation at each stage | — | — | — |
|  |  | *(c)* Consider use of a flow diagram | — | — | — |
| *Descriptive data* | **14*** | *(a)* Give characteristics of study participants (eg demographic, clinical, social) and information on exposures and potential confounders | X | Table 1 |  |
|  |  | *(b)* Indicate number of participants with missing data for each variable of interest | X | Page 2 | Volunteers with missing data were excluded |
|  |  | *(c)* Cohort study—Summarise follow-up time (eg, average and total amount) |  |  |  |
| *Outcome data* | **15*** | Cohort study—Report numbers of outcome events or summary measures over time | X | Table 2-4 and Figure 1 |  |
|  |  | Case-control study—Report numbers in each exposure category, or summary measures of exposure |  |  |  |
|  |  | Cross-sectional study—Report numbers of outcome events or summary measures |  |  |  |
| *Main results* | **16** | *(a)* Give unadjusted estimates and, if applicable, confounder-adjusted estimates and their precision (eg, 95% confidence interval). Make clear which confounders were adjusted for and why they were included | X | Table 2-4 and Figure 1 |  |
|  |  | *(b)* Report category boundaries when continuous variables were categorized | X | Table 2-4 and Figure 1 |  |
|  |  | *(c)* If relevant, consider translating estimates of relative risk into absolute risk for a meaningful time period |  |  |  |
| *Other analyses* | **17** | Report other analyses done—eg analyses of subgroups and interactions, and sensitivity analyses | X |  |  |
| **Discussion** |  |  |  |  |  |
| *Key results* | **18** | Summarise key results with reference to study objectives | X | Pages 6-8 |  |
| *Limitations* | **19** | Discuss limitations of the study, taking into account sources of potential bias or imprecision. Discuss both direction and magnitude of any potential bias | X | Pages 6-8 |  |
| *Interpretation* | **20** | Give a cautious overall interpretation of results considering objectives, limitations, multiplicity of analyses, results from similar studies, and other relevant evidence | X | Pages 6-8 |  |
| *Generalisability* | **21** | Discuss the generalisability (external validity) of the study results | X | Pages 6-8 |  |
| **Other information** |  |  |  |  |  |
| *Funding* | **22** | Give the source of funding and the role of the funders for the present study and, if applicable, for the original study on which the present article is based | X | Conflict of Interest |  |
| **TOTAL SCORE: 20** |  |  |  |  |  |

| **STROBE Statement—checklist of items that should be included in reports of observational studies** |  |  |  |  |  |
| --- | --- | --- | --- | --- | --- |
|  |  |  |  |  |  |
| **Article:** Macronutrients, Diet Quality, and Frailty in Older Men | **Year:** 2014 |  |  |  |  |
| **Authors:**  Shikany et a., |  |  |  |  |  |
|  |  |  |  |  |  |
|  | **Item No** | **Recommendation** | **Reported** | **Page (paragraph [line])** | **Note** |
| **Title and abstract** | **1** | *(a)* Indicate the study’s design with a commonly used term in the title or the abstract | X | Page 695 |  |
|  |  | *(b)* Provide in the abstract an informative and balanced summary of what was done and what was found | X | Page 695 |  |
| **Introduction** |  |  |  |  |  |
| *Background/rationale* | **2** | Explain the scientific background and rationale for the investigation being reported | X | Pages 695-696 |  |
| *Objectives* | **3** | State specific objectives, including any prespecified hypotheses | X | Pages 695-696 |  |
| **Methods** |  |  |  |  |  |
| *Study design* | **4** | Present key elements of study design early in the paper | X | Page 696 |  |
| *Setting* | **5** | Describe the setting, locations, and relevant dates, including periods of recruitment, exposure, follow-up, and data collection | X | Page 696 |  |
| *Participants* | **6** | *(a)* Cohort study—Give the eligibility criteria, and the sources and methods of selection of participants. Describe methods of follow-up | X | Pages 696-697 (2) |  |
|  |  | Case-control study—Give the eligibility criteria, and the sources and methods of case ascertainment and control selection. Give the rationale for the choice of cases and controls |  |  |  |
|  |  | Cross-sectional study—Give the eligibility criteria, and the sources and methods of selection of participants |  |  |  |
|  |  | *(b)* Cohort study—For matched studies, give matching criteria and number of exposed and unexposed |  |  |  |
|  |  | Case-control study—For matched studies, give matching criteria and the number of controls per case |  |  |  |
| *Variables* | **7** | Clearly define all outcomes, exposures, predictors, potential confounders, and effect modifiers. Give diagnostic criteria, if applicable | X | Pages 696-697 |  |
| *Data sources/ measurement* | **8*** | For each variable of interest, give sources of data and details of methods of assessment (measurement). Describe comparability of assessment methods if there is more than one group | X | Pages 696-697 |  |
| *Bias* | **9** | Describe any efforts to address potential sources of bias | — | — | This information was not mentioned |
| *Study size* | **10** | Explain how the study size was arrived at | — | — | This information was not mentioned |
| *Quantitative variables* | **11** | Explain how quantitative variables were handled in the analyses. If applicable, describe which groupings were chosen and why | X | Pages 696-697 |  |
| *Statistical methods* | **12** | *(a)* Describe all statistical methods, including those used to control for confounding | X | Page 697 |  |
|  |  | *(b)* Describe any methods used to examine subgroups and interactions | X | Page 697 |  |
|  |  | *(c)* Explain how missing data were addressed | X | Page 697 |  |
|  |  | *(d)* Cohort study—If applicable, explain how loss to follow-up was addressed | X | Page 696 |  |
|  |  | Case-control study—If applicable, explain how matching of cases and controls was addressed |  |  |  |
|  |  | Cross-sectional study—If applicable, describe analytical methods taking account of sampling strategy |  |  |  |
|  |  | *(e)* Describe any sensitivity analyses |  |  |  |
| **Results** |  |  |  |  |  |
| *Participants* | **13*** | *(a)* Report numbers of individuals at each stage of study—eg numbers potentially eligible, examined for eligibility, confirmed eligible, included in the study, completing follow-up, and analysed | X | Page 697 |  |
|  |  | *(b)* Give reasons for non-participation at each stage | X | Page 697 |  |
|  |  | *(c)* Consider use of a flow diagram | — | — | This information was not mentioned |
| *Descriptive data* | **14*** | *(a)* Give characteristics of study participants (eg demographic, clinical, social) and information on exposures and potential confounders | X | Table 1 |  |
|  |  | *(b)* Indicate number of participants with missing data for each variable of interest | — | — | This information was not mentioned |
|  |  | *(c)* Cohort study—Summarise follow-up time (eg, average and total amount) | X | Pages 696-697 |  |
| *Outcome data* | **15*** | Cohort study—Report numbers of outcome events or summary measures over time | X | Table 2-3 |  |
|  |  | Case-control study—Report numbers in each exposure category, or summary measures of exposure |  |  |  |
|  |  | Cross-sectional study—Report numbers of outcome events or summary measures |  |  |  |
| *Main results* | **16** | *(a)* Give unadjusted estimates and, if applicable, confounder-adjusted estimates and their precision (eg, 95% confidence interval). Make clear which confounders were adjusted for and why they were included | X | Table 2-3 |  |
|  |  | *(b)* Report category boundaries when continuous variables were categorized | X | Page 697 |  |
|  |  | *(c)* If relevant, consider translating estimates of relative risk into absolute risk for a meaningful time period |  |  |  |
| *Other analyses* | **17** | Report other analyses done—eg analyses of subgroups and interactions, and sensitivity analyses | X | Tables 2-3 |  |
| **Discussion** |  |  |  |  |  |
| *Key results* | **18** | Summarise key results with reference to study objectives | X | Pages 698-701 |  |
| *Limitations* | **19** | Discuss limitations of the study, taking into account sources of potential bias or imprecision. Discuss both direction and magnitude of any potential bias | X | Pages 698-701 |  |
| *Interpretation* | **20** | Give a cautious overall interpretation of results considering objectives, limitations, multiplicity of analyses, results from similar studies, and other relevant evidence | X | Pages 698-701 |  |
| *Generalisability* | **21** | Discuss the generalisability (external validity) of the study results | X | Pages 698-701 |  |
| **Other information** |  |  |  |  |  |
| *Funding* | **22** | Give the source of funding and the role of the funders for the present study and, if applicable, for the original study on which the present article is based | X | Funding |  |
| **TOTAL SCORE: 20** |  |  |  |  |  |

| **STROBE Statement—checklist of items that should be included in reports of observational studies** |  |  |  |  |  |
| --- | --- | --- | --- | --- | --- |
|  |  |  |  |  |  |
| **Article:** Macronutrients Intake and Incident Frailty in Older Adults: A Prospective Cohort Study | **Year:** 2016 |  |  |  |  |
| **Authors:**  Sandoval-Insausti et al., |  |  |  |  |  |
|  |  |  |  |  |  |
|  | **Item No** | **Recommendation** | **Reported** | **Page (paragraph [line])** | **Note** |
| **Title and abstract** | **1** | *(a)* Indicate the study’s design with a commonly used term in the title or the abstract | X | Pages 1-2 |  |
|  |  | *(b)* Provide in the abstract an informative and balanced summary of what was done and what was found | X | Pages 1-2 |  |
| **Introduction** |  |  |  |  |  |
| *Background/rationale* | **2** | Explain the scientific background and rationale for the investigation being reported | X | Page 2 |  |
| *Objectives* | **3** | State specific objectives, including any prespecified hypotheses | X | Page 2 |  |
| **Methods** |  |  |  |  |  |
| *Study design* | **4** | Present key elements of study design early in the paper | X | Page 3 |  |
| *Setting* | **5** | Describe the setting, locations, and relevant dates, including periods of recruitment, exposure, follow-up, and data collection | X | Page 3 |  |
| *Participants* | **6** | *(a)* Cohort study—Give the eligibility criteria, and the sources and methods of selection of participants. Describe methods of follow-up | X | Page 3 |  |
|  |  | Case-control study—Give the eligibility criteria, and the sources and methods of case ascertainment and control selection. Give the rationale for the choice of cases and controls |  |  |  |
|  |  | Cross-sectional study—Give the eligibility criteria, and the sources and methods of selection of participants |  |  |  |
|  |  | *(b)* Cohort study—For matched studies, give matching criteria and number of exposed and unexposed |  |  |  |
|  |  | Case-control study—For matched studies, give matching criteria and the number of controls per case |  |  |  |
| *Variables* | **7** | Clearly define all outcomes, exposures, predictors, potential confounders, and effect modifiers. Give diagnostic criteria, if applicable | X | Pages 3-4 |  |
| *Data sources/ measurement* | **8*** | For each variable of interest, give sources of data and details of methods of assessment (measurement). Describe comparability of assessment methods if there is more than one group | X | Pages 3-4 |  |
| *Bias* | **9** | Describe any efforts to address potential sources of bias | — | — | This information was not mentioned |
| *Study size* | **10** | Explain how the study size was arrived at | — | — | This information was not mentioned |
| *Quantitative variables* | **11** | Explain how quantitative variables were handled in the analyses. If applicable, describe which groupings were chosen and why | X | Pages 3-5 |  |
| *Statistical methods* | **12** | *(a)* Describe all statistical methods, including those used to control for confounding | X | Pages 4-5 |  |
|  |  | *(b)* Describe any methods used to examine subgroups and interactions | X | Pages 4-5 |  |
|  |  | *(c)* Explain how missing data were addressed | X | Page 3 | Volunteers with missing data were excluded |
|  |  | *(d)* Cohort study—If applicable, explain how loss to follow-up was addressed |  |  |  |
|  |  | Case-control study—If applicable, explain how matching of cases and controls was addressed |  |  |  |
|  |  | Cross-sectional study—If applicable, describe analytical methods taking account of sampling strategy |  |  |  |
|  |  | *(e)* Describe any sensitivity analyses | X | Page 5 |  |
| **Results** |  |  |  |  |  |
| *Participants* | **13*** | *(a)* Report numbers of individuals at each stage of study—eg numbers potentially eligible, examined for eligibility, confirmed eligible, included in the study, completing follow-up, and analysed | X | Pages 3 and 5 |  |
|  |  | *(b)* Give reasons for non-participation at each stage | X | Page 5 |  |
|  |  | *(c)* Consider use of a flow diagram | — | — | This information was not mentioned |
| *Descriptive data* | **14*** | *(a)* Give characteristics of study participants (eg demographic, clinical, social) and information on exposures and potential confounders | X | Supplementar material |  |
|  |  | *(b)* Indicate number of participants with missing data for each variable of interest | X | Pages 3 and 5 | Volunteers with missing data were excluded |
|  |  | *(c)* Cohort study—Summarise follow-up time (eg, average and total amount) | X | Page 5 |  |
| *Outcome data* | **15*** | Cohort study—Report numbers of outcome events or summary measures over time | X | Page 5 |  |
|  |  | Case-control study—Report numbers in each exposure category, or summary measures of exposure |  |  |  |
|  |  | Cross-sectional study—Report numbers of outcome events or summary measures |  |  |  |
| *Main results* | **16** | *(a)* Give unadjusted estimates and, if applicable, confounder-adjusted estimates and their precision (eg, 95% confidence interval). Make clear which confounders were adjusted for and why they were included | X | Tables 1 and 2 |  |
|  |  | *(b)* Report category boundaries when continuous variables were categorized | X | Pages 5-9 |  |
|  |  | *(c)* If relevant, consider translating estimates of relative risk into absolute risk for a meaningful time period |  |  |  |
| *Other analyses* | **17** | Report other analyses done—eg analyses of subgroups and interactions, and sensitivity analyses | X | Supplementar material |  |
| **Discussion** |  |  |  |  |  |
| *Key results* | **18** | Summarise key results with reference to study objectives | X | Pages 9-12 |  |
| *Limitations* | **19** | Discuss limitations of the study, taking into account sources of potential bias or imprecision. Discuss both direction and magnitude of any potential bias | X | Pages 11-12 |  |
| *Interpretation* | **20** | Give a cautious overall interpretation of results considering objectives, limitations, multiplicity of analyses, results from similar studies, and other relevant evidence | X | Pages 9-12 |  |
| *Generalisability* | **21** | Discuss the generalisability (external validity) of the study results | X | Pages 9-12 |  |
| **Other information** |  |  |  |  |  |
| *Funding* | **22** | Give the source of funding and the role of the funders for the present study and, if applicable, for the original study on which the present article is based | X | Funding |  |
| **TOTAL SCORE: 20** |  |  |  |  |  |
